# Unraveling human protein interaction networks underlying co-occurrences of diseases and pathological conditions

**DOI:** 10.1186/1479-5876-12-99

**Published:** 2014-04-14

**Authors:** Hyojung Paik, Hyoung-Sam Heo, Hyo-jeong Ban, Seong Beom Cho

**Affiliations:** 1 Division of Bio-Medical Informatics, Center for Genome Science, National Institute of Health, OHTAC, 187 Osongsaengmyeong2(i)-ro, Gangoe-myeon, Cheongwon-gun, ChoongchungBuk-do, South Korea; 2 Department of Pediatrics and the Department of Medicine, Stanford University School of Medicine, 94305 Stanford, CA, USA; 3 Lucile Packard Children’s Hospital, 725 Welch Road, 94304 Palo Alto, CA, USA; 4 Division of Molecular and Life Sciences, Hanyang University, Gyeonggi-do 425-791, Ansan, South Korea

**Keywords:** Comorbidity, Protein–protein interaction, Attack tolerance

## Abstract

**Background:**

Human diseases frequently cause complications such as obesity-induced diabetes and share numbers of pathological conditions, such as inflammation, by dysfunctions of common functional modules, such as protein–protein interactions (PPIs).

**Methods:**

Our developed pipeline, ICod (Interaction analysis for disease Comorbidity), grades similarities between pairs of disease-related PPIs including comorbid diseases and pathological conditions. ICod displayed a disease similarity network consisting of nodes of disease PPIs and edges of similarity value. As a proof of concept, eight complex diseases and pathological conditions, such as type 2 diabetes, obesity, inflammation, and cancers, were examined to discover whether PPIs shared between diseases were associated with comorbidities.

**Results:**

By comparing Medicare reports of disease co-occurrences from 31 million patients, the disease similarity network shows that PPIs of pathological conditions, including insulin resistance, and inflammation, overlap significantly with PPIs of various comorbid diseases, including diabetes, obesity, and cancers (*p* < 0.05). Interestingly, maintaining connectivity between essential genes was more drastically perturbed by removing a node of a disease-related gene rather than a pathological condition-related gene, such as one related to inflammations.

**Conclusion:**

Thus, PPIs of pathological symptoms are underlying functional modules across diseases accompanying comorbidity phenomena, whereas they contribute only marginally to maintaining interactions between essential genes.

## Background

Most diseases are the result of the collapse of cellular processes together with interaction networks among components of the genome, proteome, and metabolome, and these perturbed components are likely to be linked with other diseases [1]. Indeed, disease comorbidities such that the onset of one disease increases the likelihood of the development of other diseases were correlated with the breakdown of common functional modules of disease pairs, such as metabolic and cellular networks [2,3]. Therefore, exploring the biological network between diseases, such as protein–protein interactions (PPIs) of chronic diseases and complications, might give us a more detailed understanding of disease comorbidity and the functional differences between complex diseases.

A number of previous attempts at “network analysis” of diseases have revolutionized our knowledge about the relationships between human diseases and comorbidity [1,2,4-6]. For instance, disease-related genetic mutations of genes tend to be peripheral nodes of the essential network, while somatic mutations of genes related to cancers were central nodes [1]. However, pathological phenotypes linked with comorbid diseases and complications remain unclear in the graph-theoretic frame. While distinct diseases share pathological symptoms and various comorbidity patterns, such as inflammations commonly associated with obesity and diabetes, a network model to depict sharing of conditions between diseases remains uncertain. In addition, network models to portray differences between diseases and pathological symptoms leading to severe (or minor) abnormalities of vital functions have been scarcely addressed, whereas distinct mortality issues have been highlighted among cancer-like diseases and pathological symptoms [7,8].

Here, we designed a novel method, ICod, to build similarity networks among PPIs of disease and pathological conditions to address relationships between comorbid disease pairs and pathological symptoms. While there are various patterns of disease comorbidity, we focused obesity as one of leading risk factors contributing to the overall burden of disease worldwide [9]. Among various obesity related complications, we selected seven diseases and pathological symptoms, which have been remarked as obesity related diseases and manifestations [9,10]. Thus, the disease studied are obesity, type 2 diabetes mellitus (T2DM), breast cancer, colon cancer, and prostate cancer, and pathological symptoms are inflammation, insulin resistance, and immune response. The main assumption of ICod is that dysfunctions of common protein interactions between diseases might lead to disease comorbidities. To evaluate phenomic associations between network similarities of diseases and comorbidity patterns, disease co-occurrences in a human population were also interrogated using onset co-occurrence relationships based on 31 million patients [11] (http://hudine.neu.edu/). Furthermore, we address the structural importance of disease- and pathological condition-related genes in maintaining connectivity in the network of essential genes to suggest distinct network models for the dysfunction degrees under diseases or pathological symptoms including inflammation. The attack tolerance of the essential network was determined by measuring alterations of network diameter following removal of disease- and pathological symptom-related essential genes, respectively. The network diameter, defined as the average length of the shortest paths between any two nodes in a network, represents the ability to communicate between any two nodes within the network [12].

## Materials and methods

### ICod: Similarity of disease- and pathological condition-related PPIs

ICod used the following measures to grade the disease–disease similarities based on disease-related PPIs. The PPI network modules of each disease were explored using the disease-related genes in our datasets. A disease-related network was produced based on the first neighboring nodes of each disease-related gene. The distance between each pair of disease-related genes was calculated based on the shortest path in the integrated human PPI network. The distances were normalized by transforming them using the formula of Perlman *et al.*[13]. The transformed distances of all the protein pairs in the two disease-related networks were used to compute the proportion of overlap by considering the size of each corresponding network. The detailed equation is:

(1) $$\mu \left(\mathrm{NE}T_{i},\mathrm{NE}T_{j}\right)=\frac{\sum_{D\left(P_{n},P_{m}\right)\leq C}S\left(P_{n},P_{m}\right)}{\sum_{P_{n}\in \mathrm{NE}T_{i},P_{m}\in \mathrm{NE}T_{j}}S\left(P_{n},P_{m}\right)}$$

where *NET*_*i*_ and *NET*_*j*_ denote the networks related to diseases *i* and *j*, respectively, D(*p*_*n*_, *p*_*m*_) is the length of the shortest path between protein *p*_*n*_ and *p*_*m*_, and *S*(*p*_*n*_, *p*_*m*_) are the transformed distances between the networks based on the definition provided by Perlman *et al.*[13]:

(2) $$S\left(p_{n},p_{m}\right)=A\;\exp\left(–\mathrm{bD}\left(p_{n},p_{m}\right)\right)$$

We used *A* = 0.9 and *b* = 1, as recommended by Perlman *et al.*[13]. *C* is the threshold of D(*p*_*n*_, *p*_*m*_), which indicates sufficient proximity between two proteins. We used *C* = 0 to consider directly overlapping proteins in two disease-related networks. Thus, μ(*NET*_*i*_, *NET*_*j*_) represents the normalized proportion of the overlap based on the overall size of the networks. The statistical significance of μ(*NET*_*i*_, *NET*_*j*_) was measured as the *p* value based on the background distribution of μ in 1000 randomly permuted tests. With identical manner, we also determined similarities between pathological conditions.

### Preparation of datasets

#### List of seed genes

We used 317 genes related to five diseases (obesity, T2DM, breast cancer, prostate cancer, and colon cancer) and three pathological conditions (inflammation, immune response, and insulin resistance). These genes were collected from the public resource, GeneCards [14], which was searched by using related keywords such as “breast cancer”, “malignant neoplasm of breast”, “T2DM,” and “insulin resistance” (Additional file 1: Table S1). All of disease related keywords were manually selected from the results of concept ID search on the largest biomedical terminology database, UMLS (Unified Medical Language System) [15]. In case of immune response, we combined results of immune disorder to comprising immune response related disease symptoms.

#### Protein–protein interaction (PPI) network

We integrated various well-known resources to prepare human PPI networks: the Human Protein Reference Database (HPRD) [16]; BioGrid (the Biological General Repository for Interaction Datasets) [17]; IntAct [18]; the Molecular INTeraction database (MINT) [19]; and the Database of Interacting Proteins (DIP) [20]. To produce valid PPI networks, we only used protein interactions with physical evidence; i.e., those with Proteomics Standard Initiative —Molecular Interactions (PSI-MI) codes, such as physical interactions (MI: 0218), direct interactions (MI: 0407), and physical associations (MI: 0915).

## Results

### Overview of ICod pipeline

**Figure 1:**
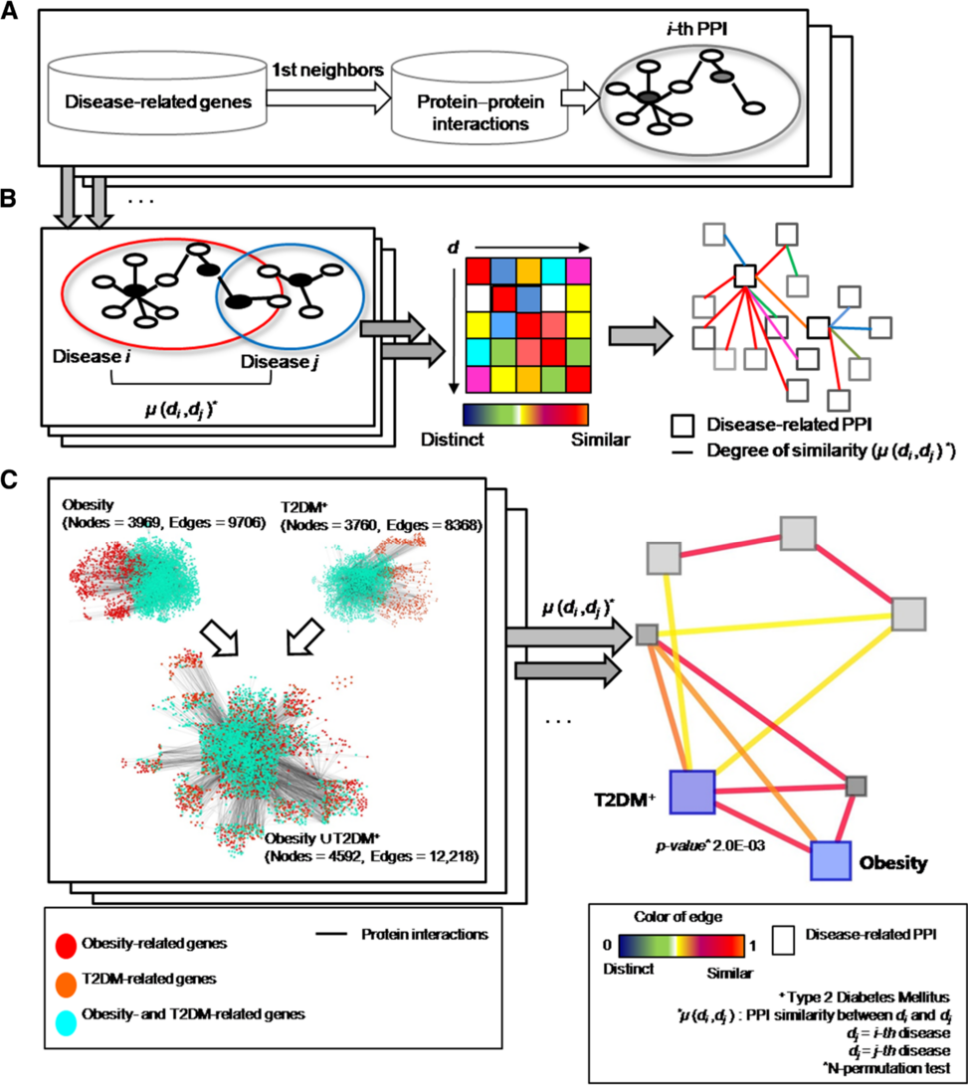
**Overview of ICod: network-based disease–disease similarity analysis. A**. Generation of disease-related protein–protein interaction (PPI) networks based on seed genes. **B**. Degree of network similarity (μ) determines disease–disease similarity by analyzing the proportion of intersections between disease-related PPIs. **C**. Detailed example of grading PPI-based similarity between type 2 diabetes mellitus (T2DM) and obesity using ICod.

The starting point of ICod is the generation of seed genes related to diseases or pathological conditions. A search of GeneCards was made using 33 keywords, and a total of 317 genes related to five diseases (obesity, T2DM, prostate cancer, breast cancer, and colon cancer) and three pathological symptoms (inflammation, insulin resistance, and immune response) were prepared [21] (Additional file 2: Table S2). Using these seed genes, subnetworks of diseases of interest and pathological conditions were prepared by exploring nearest neighbors among integrated human PPIs over 11 K of nodes and 113 K of edges from five different databases [16-20] (Figure 1A). Then, ICod computes disease–disease similarities considering degrees of overlap between disease- or pathological condition-related PPIs to produce a disease similarity network graphically, as depicted in Figure 1B. The disease similarity network consists of nodes for disease PPIs and edges for degree of similarity between the *i-*th and *j-*th PPIs (μ(*d*_*i*_, *d*_*j*_)). The detailed equations for μ(*d*_*i*_, *d*_*j*_) are described in the Methods section. Figure 1C shows an example ICod pipeline to determine similarity, μ, between obesity- and T2DM-related PPIs after building subnetworks consisting of 3,969 obesity genes and 3,760 T2DM genes. For the statistical significance, *p* value of μ(*d*_*obesity*_, *d*_*T2DM*_ ) was assessed using random permutation tests.

### PPI similarity and comorbidity patterns among diseases and pathological conditions

**Figure 2:**
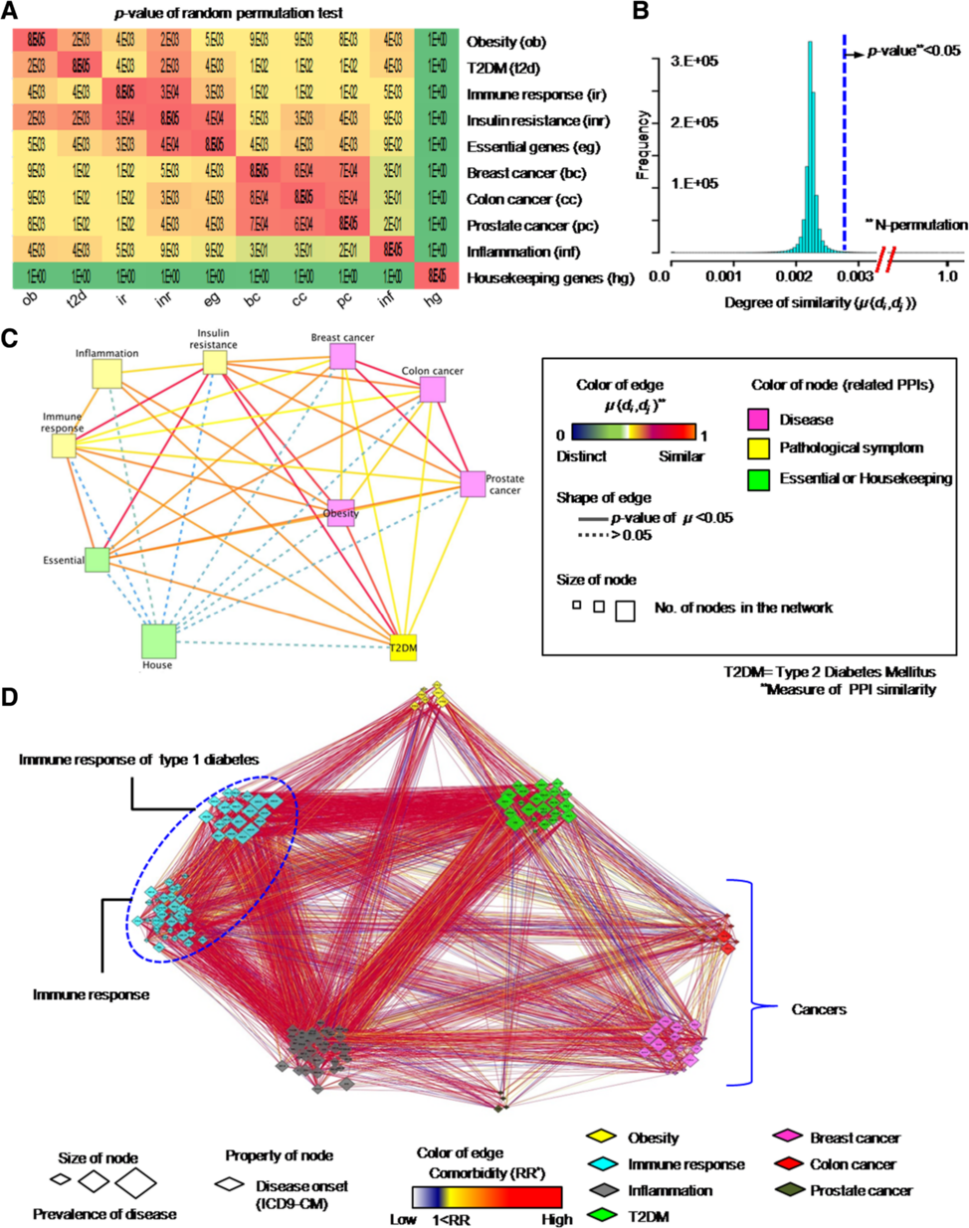
**Similarity of disease-related PPIs and disease-comorbidity network. A**. *p* values of disease–disease PPI similarity (μ(*d*_*i*_, *d*_*j*_)) matrix computed by ICod. **B**. Generated background distribution of similarity values via random permutation tests. **C**. Disease similarity network consisting of nodes of disease PPIs and edges of similarity values. In the present study, five diseases (T2DM, obesity, prostate cancer, colon cancer, and breast cancer) and three pathological conditions (immune response, inflammation, and insulin resistance) were analyzed. Housekeeping and essential gene-related PPIs were regarded as a control set of nondisease conditions. **D**. Phenomic-level disease and pathological symptom co-occurring networks based on US Medicare data. Because the clinical records utilized diagnostic codes from ICD9-CM (International Classification of Diseases, 9th Revision, Clinical Modification) to determine the disease or pathological state of patients, we manually assigned the relevant names of diseases, such as prostate cancer and obesity, to the reported codes of ICD9-CM.

Figure 2A shows the *p* values of the disease–disease PPI similarity (μ(*d*_*i*_, *d*_*j*_)) matrix calculated by ICod. Housekeeping and essential gene networks, as controls for nondisease networks, were built from 2,164 seed genes and their neighbor nodes (2064 for housekeeping and 115 for essential genes) from previous attempts [22,23]. Figure 2B shows the background distribution of similarity values to compute the *p* values of the disease–disease similarity value, μ. Based on these permutation test results, inflammation, insulin resistance, and immune response show high similarity with multiple diseases (*p* < 0.05, Figure 2A).

As shown in Figure 2C, the similarity between obesity and T2DM is significantly high (*p* = 2.27E–03) and pathological conditions significantly overlapped with various diseases. Disease- and pathological condition - PPIs, except inflammation, are significantly similar to the essential network (*p* < 0.05). Thus, insulin resistance- and immune response-related PPIs were commonly incorporated in various disease and essential gene networks.

Figure 2D depicts onset co-occurrence of disease groups interrogated from previous attempts utilizing the medical records of 31 million patients [11]. Using the network frame, we displayed statistical significances of disease co-occurrences (i.e., comorbidities) based on relative risk values [11]. In the presented comorbidity network, nodes mean disease onsets and edges are relative risk values between nodes. As displayed in Figure 2D, inflammation-related symptoms (gray nodes) are closely associated with the onset of obesity (yellow nodes) and T2DM (green nodes).

As Figure 2C and D depict, comorbidities showed similar tendencies to PPI similarity networks of diseases. The network analysis presented supports the hypothesis that the collapse of common PPIs between disease and pathological symptoms were closely associated with comorbidity.

### Topological role of disease- and pathological condition-related genes for essential interactions

Irrespective of mortality rate, pathological symptom-related PPIs overlap significantly with various diseases including cancers and even networks of essential genes. Using the measure of node degree (i.e., number of nearest neighbors), a previous network-based attempt suggested that disease-related genes were peripheral nodes in the essential network [1]. Nevertheless, cancer-like diseases are major causes of death in the world [7], although models showing a severe impact on essential gene networks remain imprecise. Here, except for node degree, we compared topological importance between disease- and pathological condition-related nodes through measuring the collapsed connectivity of the essential network by elimination of disease- or symptom-related essential genes.

**Figure 3:**
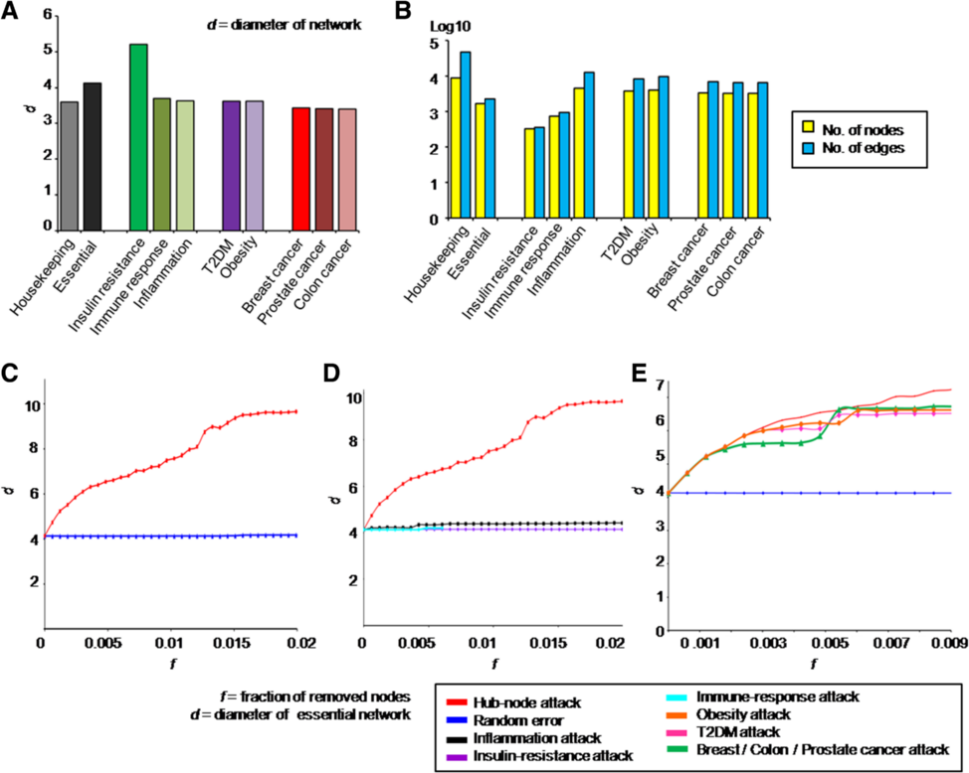
**Disease- and pathological condition-related nodes in the essential network. A**. Bar charts of network diameters for five diseases, three pathological conditions, and nondisease gene-related PPIs (i.e., housekeeping and essential genes). **B**. Numbers of edges and nodes in the five disease- and three pathological conditions-related PPIs in log scale. In addition, the numbers of nodes and edges in the housekeeping and essential networks are also presented. **C**, **D**, **E**. Alteration of the diameter of the essential network by removing nodes. As a scale-free network, the essential network was robust against random errors (blue line in panel **C**), but was vulnerable to high-degree node attacks (red line in **C**). While an attack of pathological conditions showed random error-like effects on the essential network (cyan, black and violet lines in **D**), attacks on disease-related essential genes caused dramatic perturbations to the linkages within the essential network (green, pink and orange lines in **E**).

By the definition of network diameter (mean of shortest paths between all pairs of nodes in a network) [12], a pattern of increasing the diameter by removing a node denotes the breakage of network links and the vital role of the removed node in maintaining information flows in the network. According to Figure 3A and B, the network diameters of disease PPIs, pathological condition PPIs, housekeeping PPIs, and essential PPIs indicate a diverse density of connectivity within each network unrelated to the number of nodes or edges. Because the PPI network of human essential genes is a scale-free network, it is robust against network errors while it is vulnerable against attacks on high-degree nodes (Figure 3C). “Network attack” means sequential removal of nodes according to node rank (i.e., a hub-node attack), whereas “network error” (i.e., random error) means withdrawing a node randomly.

Interestingly, alteration of the essential network diameter by attacks on the pathological condition-related node was negligible (Figure 3D), whereas the connectivity of the essential network collapsed dramatically on removal of a disease-related essential gene (Figure 3E). The connectivity of the essential network (diameter 4.13) was dramatically perturbed even under attack of a small fraction of nodes related to diseases (0.1% of nodes in Figure 3E), such as T2DM, cancers, and obesity. However, attacks on a larger fraction of nodes related to pathological symptoms, including insulin resistance, inflammation, and immune response, showed subtle effects in truncating interactions among essential genes (Figure 3D). Based on these distinct topological roles of disease- and symptom-related nodes in the essential network, we suggest that disease-related nodes are vital nodes of information flow in the essential network, whereas nodes of pathological symptom play a less pivotal role.

## Discussion

In summary, using ICod, we determined relationships among five diseases (prostate cancer, breast cancer, colon cancer, T2DM, and obesity), three pathological conditions (inflammation, insulin resistance, and immune response), and the essential gene network. As expected from pathological symptoms in complex diseases sharing common phenotypic signals including inflammation, the results of ICod support our knowledge at the network level. The pathological condition network is closely associated with various disease networks. Our findings are the first attempt at uncovering the differences in topological role between each disease- and symptom-related network within the essential gene network using analysis of attack tolerance. Although PPIs of pathological conditions significantly overlapped with disease PPIs, the patterns of collapsing the essential network by removing the condition-related nodes were clearly distinct from attacks on disease-related nodes. While our network analysis covered partial sets of human diseases and symptoms, our conceptual approach successfully modeled functional roles of pathological states in disease etiology and maintenance of the essential network. Typical pathological symptoms, such as inflammation and immune responses, are widely spread mechanisms behind complex diseases with subtle impacts that can cause severe dysfunctions of the essential network.

Since our network model focused topological similarity, our method suggested network relationships between disease pairs, or disease-pathological symptoms without causal understandings and functional significance. To address network related functional impact (i.e., complete node removal and partial mutation), Zhong *et al.* attempted computational and experimental validation using Yeast-Two-Hybrid system (Y2H) [24]. In our previous study, we analyzed gene expression patterns in diet induced obese mice [25]. Interestingly, diet-induced obese mice displayed differentially expressed genes, which were related inflammation, immune response and insulin resistance as we suggested our network similarity analysis. While our previous work suggested enriched functional signatures under induced obesity condition without node-removal effect, significantly depict obesity derived pathological phenotype in time-resolving frame. Based on theses attempts, we suggest an approach combining Zhong *et al*’ s Y2H and time-resolving frame of ours for further functional understanding. Owing to the utilization of model organisms, Zhang *et al.* and our mouse data analysis give us limited understandings to depict underlying mechanisms of human diseases. Thus, as we conducted in our previous attempt [26], large-scale human cohort based analysis might shed light shared genetic and functional features, which lead disease comorbidity.

While cancers have shown high mortality rates [7], obesity and T2DM have low attributes for viability issues. In stark contrast with our expectation, topological roles for the interactions within the essential gene network are homogeneous between lethal diseases (cancers) and other chronic diseases. Therefore, further study is necessary on the associations between disease mortality and aspects of network structure, such as “bottleneckness” [27]. In addition, our keyword-based approach to preparing disease and pathological symptom related genes were introduced for the proof-of-concept. Thus, it is necessary for advanced validation of disease related genes using various approaches, such as scrutinizing gene expression databases [28].

As shown in our network analysis of disease PPI similarity and US Medicare data, disease onset and comorbidity are closely associated with the breakage of common functional modules. Complex diseases and pathological conditions share molecular mechanisms such as PPIs, whereas mortalities are heterogeneous. We distinguished network models of complex diseases and pathological conditions using our analysis of attack tolerance of the essential network to find the different impacts on mortality issues.

One of our contributions is computing quantitative degree of overlapping between disease PPIs involving across similarity among diseases and related clinical manifestations considering connectivity of compared PPI pairs (Figure 2). Since ICod utilized public repository of PPI networks and list of disease related genes, our method can be a streamlined route to visualize similarity between diseases and pathological phenotypes, which are associated disease comorbidity [2]. In addition, our network-based disease similarity might present drug targets related various diseases as presented by Suthram *et al.*[29]. For example, ICod remarks a probability for the repositioning of drugs related pathological symptoms, such as inflammation, for the therapy of PPI overlapped diseases including obesity; The anti-inflammation drug, amlexanox, elevate energy expenditure and produce weigh loss in mice [30].

## Conclusion

Therefore, analysis of ICod network similarity and attack tolerance has successfully modeled existing knowledge for disease comorbidities and the co-occurrence of pathological symptoms, and we have identified perturbation models for disease-related essential genes through the network frame.

## Competing interests

There are no conflicts of interest.

## Authors’ contributions

HP built the main algorithm, designed the analysis methods, prepared artworks of figures and helped write the manuscript. HSH prepared datasets and helped write the manuscript. HB analyzed co-occurrence patterns of human diseases. SBC oversaw the overall organization of the manuscript as a corresponding author. All authors read and approved the final manuscript.

## Supplementary Material

Additional file 1: Table S1 Summary of keywords for preparing seed genes related to diseases and pathological conditions. Click here for file

Additional file 2: Table S2 List of diseases and pathological symptom related genes. Click here for file
