# Supplementary material for: Unraveling human protein interaction networks underlying co-occurrences of diseases and pathological conditions
Source: J Transl Med. 2014 Apr 14;12:99. doi: 10.1186/1479-5876-12-99 (PMC4021415; doi:10.1186/1479-5876-12-99)
Supplement: Additional file 1: Table S1 — Summary of keywords for preparing seed genes related to diseases and pathological conditions. [file 1479-5876-12-99-S1.docx]

**Additional file 1: Table S1.** Summary of keywords for preparing seed genes related diseases and pathological conditions.

| Diseases and pathological conditions | keywords |
| --- | --- |
| Obesity | obese  obesity |
| Type 2 diabetes mellitus | diabetes mellitus, type 2  diabetes mellitus, type II  non-insulin-dependent diabetes mellitus |
| Breast cancer | breast cancer  breast carcinoma  breast neoplasm  breast tumor  malignant neoplasm of breast |
| Colon cancer | colon cancer  colon carcinoma  colon neoplasm  colon tumor  colorectal cancer  colorectal carcinoma  colorectal neoplasm  colorectal tumor  malignant neoplasm of colon |
| Prostate cancer | prostate cancer  prostate carcinoma  prostate neoplasm  prostate tumor  malignant neoplasm of prostate |
| Immune response | immune response  immune disorder |
| Inflammation | inflammation |
| Insulin resistance | hyperinsulinemia  hyperglycemia  hyperinsulinism  insulin resistance  insulin sensitivity  insulin tolerance |
